# 

**Published:** 1900-06-16

**Authors:** 


					THE HOSPITAL. ? The standard of highest purity." ? Lancet, June IOtii, 1900.
CADBURY'S H !*? CADBURY'S
LASCory Western InfYrmar
No. 710>Vol. XXVIII. [J?S?-] Satdbday,
June 16, 1900.
?Subscription Terms,] pE)ICE TWOPENCE.

				

